# Robotic assisted free flap reconstruction of the scalp using the Symani^®^ surgical system

**DOI:** 10.1007/s00701-025-06501-y

**Published:** 2025-03-29

**Authors:** Jennifer Ashley Watson, Sören Könneker, Giuseppe Esposito, Luzie Hofmann, Bong-Sung Kim, Pietro Giovanoli, Nicole Lindenblatt

**Affiliations:** 1 Department of Plastic Surgery and Hand Surgery, University Hospital Zurich, Zurich, Switzerland; 2 Department of Neurosurgery, University Hospital Zurich, Zurich, Switzerland

**Keywords:** Symani^®^ Surgical System, Anterolateral thigh flap, Latissimus dorsi flap, Reconstruction, Soft tissue defect of the scalp

## Abstract

**Purpose:**

Robotic-assisted surgery currently is evolving as a new field in microsurgery with potential benefits for reconstructive surgery. The Symani^®^ Surgical System has shown feasibility in performing microsurgical anastomosis. Reconstruction of the soft tissue of the scalp is commonly necessary after brain surgery with reconstruction of the cranium using foreign materials such as PEEK (Polyether ether ketone). We describe our experience with free soft tissue transfer with a free anterolateral thigh (ALT) flap or free latissimus dorsi (LD) flap for reconstruction of the scalp using the Symani^®^ Surgical System for microsurgical anastomosis.

**Methods:**

We analyzed 6 patients with soft tissue defects of the scalp from September 2023 to January 2024 undergoing soft tissue reconstruction with a free ALT or LD flap. Robotic-assisted microsurgical anastomoses were performed using the Symani^®^ Surgical System.

**Results:**

4 male patients and 2 female patients (age 61-81 years, mean 71.8 years) were included. Mean hospital stay was 10 (8-13) days. The most common recipient vessels were the Superficial temporal artery and vein (66.7%). In other cases, we used the facial artery and vein. All arterial anastomoses were performed by using the Symani^®^ Surgical System. The mean operative time was 387 (328-48) minutes. The mean anastomosis time using the Symani^®^ Surgical System was 32.5 (19-44) minutes. No flap loss was observed. One patient suffered from a PEEK infection as a delayed post-operative complication.

**Conclusion:**

For reconstruction of soft tissue defects of the scalp, a free ALT or LD flap represents a proper treatment option. Performing microanastomosis using the Symani^®^ Surgical System is a safe technology and is leading to satisfactory outcomes.

## Introduction

**Fig. 1:**
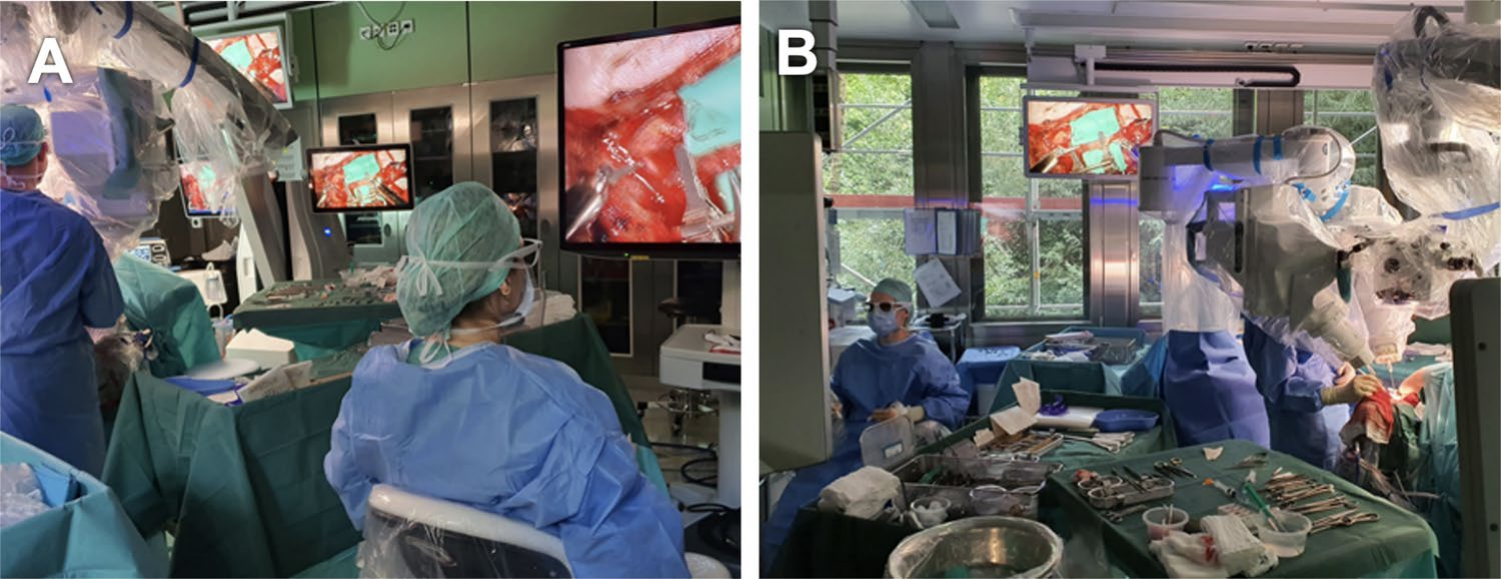
The Symani^®^ Surgical System is used with an exoscope for visualization. **A:** The surgeons use the Joysticks to direct the robotic arms without the necessity standing next to the surgical field. **B:** Via monitor the entire operating team can follow the surgery. If assistance was needed this could be easily provided by an assisting surgeon from the side of the operating table opposite to the Symani^®^ Surgical System. Figure is available in color online only

Microsurgery represents an essential technique in reconstructive surgery, especially in free flap surgery, lymphatic surgery or nerve surgery. Microsurgical skills and experience are crucial for anastomotic patency. Robotic-assisted microsurgery has recently been introduced into reconstructive surgery. The Symani^®^ Surgical System has received European conformity certification in 2019. Previously, the MUSA robot (MicroSure, Eindhoven, The Netherlands) has been introduced [14]. The Symani^®^ Surgical System is the only system that offers wristed micro-instruments so far. This robotic device is constructed with fixed joysticks, which are connected to a framework and microsurgical instruments. With the MUSA robot, the surgeon is in a fixed position. The Symani^®^ Surgical System was introduced with flexible instruments. The Symani^®^ Surgical System has already shown practicality in performing microsurgical anastomosis in reconstructive surgery as well as in emergency cases [4, 9, 11]. With its robotic arms, the system may be able to reach deeper anatomical regions through narrow pathways. Furthermore, the system also filters out hand tremors and enhances the movement of the instruments. The surgeon is in a comfortable position while movements are translated into the operative field. Valuable features like motion scaling enhance surgical precision (Fig. 1). Visualization is enabled with either a 3D visualization system or a conventional microscope (e.g. Kinevo 900 microscope (Carl Zeiss Meditec AG, Jena, Germany)). First-time transplantation of a free flap using the Symani^®^ Surgical System for microsurgical anastomosis was presented in 2023. A free ALT flap was transplanted to reconstruct a traumatic defect of the foot. An end-to-end anastomosis was performed to the medial tarsal artery and vein [8]. We have recently shown the feasibility and safety in performing micro- and supermicrosurgical anastomosis in lymphatic reconstruction with the Symani^®^ Surgical System [2, 5, 6, 11, 15].

Free flap reconstruction of the scalp is indicated in large defects with or without skull prothesis or in which other reconstructions such as local flaps are not possible or have failed. Also, in patients with insufficient tissue quality, preoperative or planned radiotherapy, full-thickness soft tissue defects and missing periosteum, free flap reconstruction is a sufficient option for wound closure. Free tissue reconstruction can provide an immediate closure of large defects and flap success rates are more than 95% [12]. When planning a reconstruction with a free flap one should always consider the patients conditions and goals in finding the ideal approach of reconstruction. There are a variety of free flap options, we report about our first cases in free flap reconstruction of the head with either a free ALT flap or a free LD flap using the Symani^®^ Surgical System for microvascular anastomosis.

Soft tissue defects of the scalp have different etiologies; neoplasms, intracranial vascular pathologies and trauma with following neurosurgical procedures are the most common. Additionally, cutaneous tumor resection is a frequent cause. Reconstruction of the cranium using foreign materials often leads to wound healing disorders. Small defects can be accomplished by primary closure or with local flaps [12]. Larger tissue defects usually require free tissue transfer. Preoperative radiation or cerebrospinal fluid leaks can complicate the reconstruction. Several reparative techniques have been described: secondary healing, skin grafts, dermal matrix, local or free flaps [7]. Skin grafts and dermal matrix require an intact periosteum. Local flaps can be used for smaller defects up to 6–8 cm^2^. The rigidity of the scalp limits the tissue mobilization, sometimes it can be overcome by weakening the galley [7]. However, in severe soft tissue defects with or without skull prothesis, the tissue must be replaced by using free flaps. A variety of free flaps are available for scalp reconstruction. Today the most popular free flaps in scalp reconstruction are the ALT or the LD flap [7]. The ALT flap has gained increasing popularity due to numerous flap modifications that can be tailored according to the needs of the defect [10]. The LD flap is indicated for extensive soft tissue defects of the scalp due to its relatively unrestricted dimensions. The ability to utilize the LD flap as a chimeric flap for simultaneous bone reconstruction of the skull further enhances its clinical advantage. In this study, we describe our experience in reconstruction of soft tissue defects of the head with a free ALT or a free LD flap using the Symani^®^ Surgical System for microsurgical anastomosis. We aimed to explore the potential of microsurgical robot-assisted anastomosis for reconstructive surgery focusing on free flap reconstruction of the scalp, thereby addressing a critical literature gap.

### Methods And materials

We conducted a retrospective mono-center study. The study was approved by the Ethics Committee of the Canton of Zurich, Switzerland (BASEC approval number 2021–02351). Between September 2023 and February 2024, 6 patients underwent a scalp reconstruction with a microsurgical procedure with the Symani^®^ Surgical System. Patients received either a free ALT flap or a free LD flap. The first two authors JAW and SK performed all surgeries. None of the surgeons had prior experience with robotic surgery. The Symani^®^ Surgical System was first introduced at the Department of Plastic Surgery and Hand Surgery in July 2021 [2, 5, 6, 11, 15]. The surgeons completed an ex vivo robotic training program, performing at least 10 successful anastomoses on artificial vessels (tubes) sized 1.0–2.0mm under supervision. The Symani^®^ Surgical System is composed of two robotic arms with a console. The console is connected to an ergonomic chair with a foot controller and two joysticks. Visualization is achieved with a 3D visualization system. The operating surgeon is seated in a comfortable position further afar from the patient. The assisting surgeon can assist with instruments and is positioned next to the operative field. The two robotic arms consist of a dilator and a needle holder with cut-off function. Stitching and knotting was performed with a needle holder on the right and a dilator on the left side with a 9-0 suture. As motion scaling, we used 7 to 10x. The arterial flap vessel was anastomosed end-to-end to the donor vessel, which was either the Superficial Temporal Artery (STA) or the Facial Artery (FA).

We included 6 patients with soft tissue defects of the scalp after brain surgery and skull reconstruction with foreign materials and following wound healing disorders, or patients with cutaneous tumors of the scalp with skull infiltration which ended in large tissue defects after excision as well as required skull prothesis. All patients were treated with a free ALT or LD flap at our clinic between September 2023 and February 2024. Personal and medical data were collected in Table 1.

**Table 1.** Clinical data

Pat ID	Sex	Age	Indication for Surgery	St.p. radiato recipient area	infection signs recipient area	Risk factors							
						Smoker	HAT	PAOD	CAD	Heart insufficiency	Kidney failure	COPD	Cancer
1	F	61	Exposed craniotomy, wound infection in the area of PEEK plastic	No	Yes	No	Yes	No	No	Yes	Yes	No	No
2	M	81	Cutaneous angiosarcoma	No	No	No	No	No	No	No	No	No	Yes
3	M	72	Pleomorphic sacroma	No	No	Yes	Yes	No	No	No	No	No	Yes
4	M	67	Exposed craniotomy, wound infection in the area of PEEK plastic	No	Yes	No	No	No	No	No	No	No	No
5	M	76	Cutaneous squamous cell carcinoma	Yes	No	No	No	No	No	No	No	No	Yes
6	F	74	Pleomorphic sacroma, metastasis	Yes	No	Yes	Yes	Yes	Yes	Yes	Yes	Yes	Yes

^CAD=coronary heart disease; COPD=chronic obstructive pulmonary disease; HTA=arterial hypertension; PEEK=Polyether ether ketone; PAOD=peripheral arterial occlusive disease.^

Preoperative examination included vascular Doppler evaluation of the flap perforators as well as of the recipient vessels. Pre- and postoperative photographic documentation was acquired. Overall, 4 patients suffered from a dermal tumor on the scalp. 3 patients were diagnoses with a dermal sarcoma; 1 patient was diagnosed with a squamous cell carcinoma. 2 of these patients underwent pre-operative radiotherapy and 2 patients underwent post-operative radiotherapy. The surgery contained R0 excision of the tumor including scalp reconstruction with a PEEK plastic in three cases. In one case R0 excision was sufficient without scalp replacement. One patient had a PEEK infection after cranial aneurysm treatment and PEEK. Another patient suffered from a PEEK infection after multiple surgical procedures based on hydrocephalus treatment. Patients with PEEK plastic and wound infections after intracranial surgery underwent initial antibiotic treatment and PEEK Re-Implantation and free flap reconstruction at the same time. Patients with cutaneous tumors and infiltration of the skull received R0 excision and reconstruction with a PEEK plastic, and free flap reconstruction. The surgery started with the definition of the size of the defect. The flap size and design were defined, and the flap was raised. One surgeon was raising the flap while the other surgeon was preparing the recipient vessels. Neurosurgeons reconstructed the skull with a PEEK plastic in the beginning of the surgery. The tissue defect was reconstructed by using either a free ALT or LD flap. As recipient vessels we used the STA (66.7%) and the FA (33.3%). All arterial anastomoses were performed by using the Symani^®^ Surgical System. Anastomosis were performed with non-absorbable interrupted sutures (Nylon 9-0). Venous anastomoses were performed by using end-to-end anastomotic devices (Coupler^®^). Intravenous perioperative antibiotic prophylaxis was applied.

## Results

**Fig. 2:**
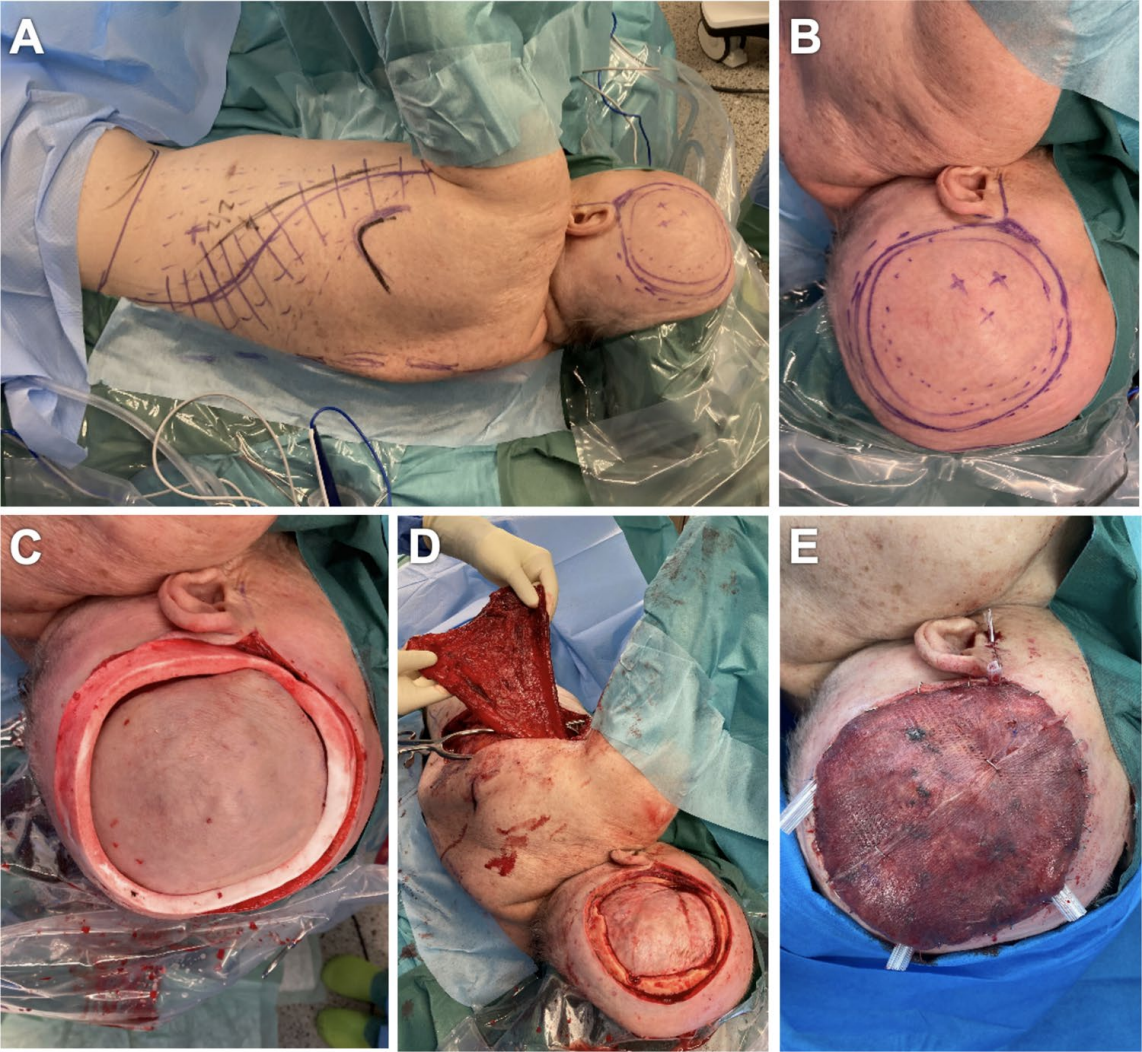
74y old patient with metastasis of a pleomorphic sarcoma of the scalp. The patient underwent neoadjuvant radiotherapy followed by R0 excision of the tumor with reconstruction of the scalp with PEEK plastic. A free latissimus dorsi flap from the left was used to reconstruct the soft tissue defect. As donor artery we used the Superficial temporal artery. **A-B:** Intra-operative view pre-excision. **C-D:** Intra-operative view of the defect after R0 excision. **E:** Latissimus dorsi flap tailoring. Figure is available in color online only

**Fig. 3:**
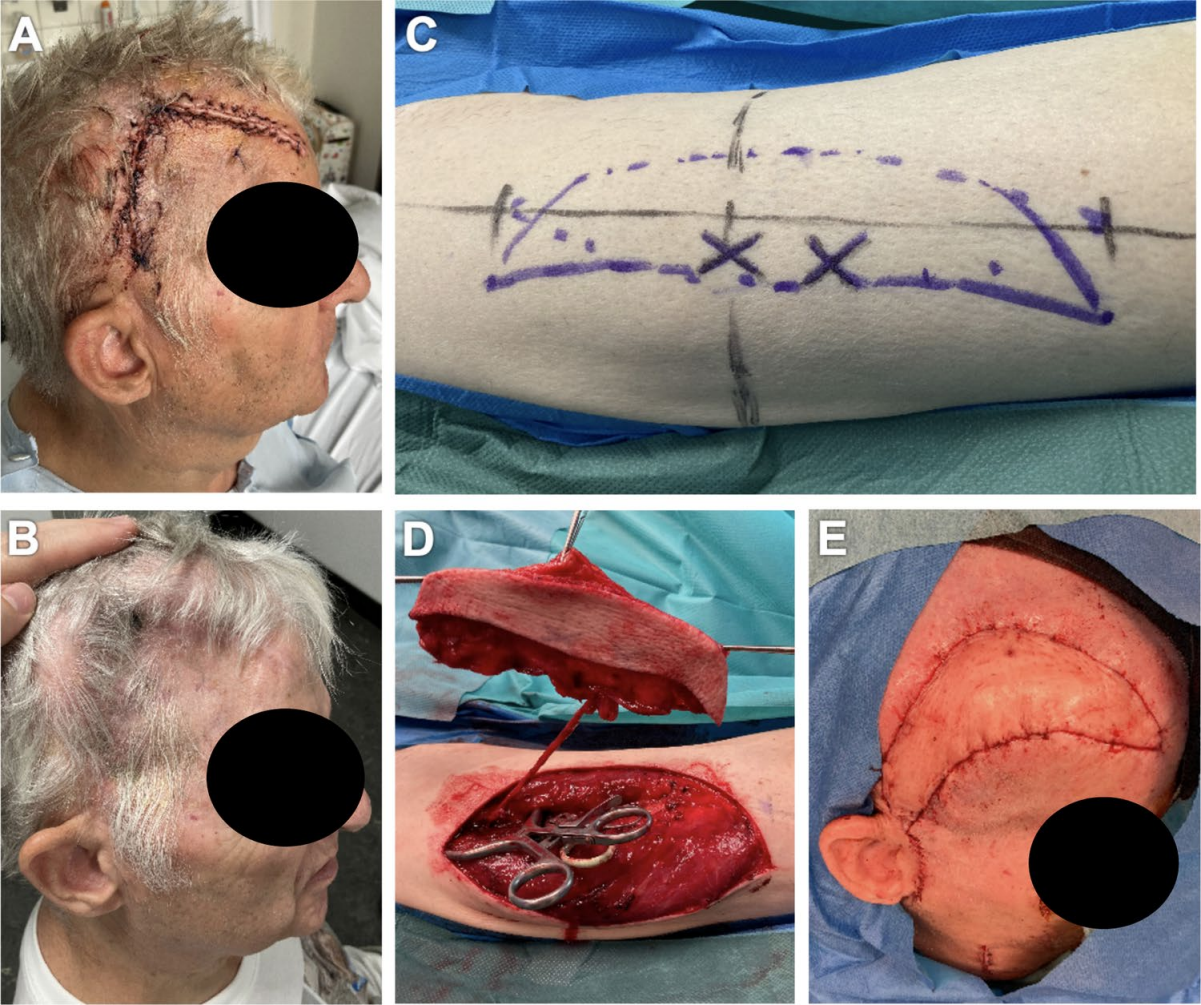
67y old patient with wound healing disorder after multiple surgical interventions regarding a Hydrocephalus malresorptivus. The patient underwent multiple scalp reconstructions with PEEK plastics. After the recent PEEK infection and explantation, PEEK Re-Implantation followed with necessary soft tissue reconstruction using a modified free ALT-flap from the right leg. Facial artery was used as donor vessel. **A-B:** Pre-operative situation with the missing skull reconstruction. **C-D:** Intra-operative flap planning. **E:** ALT flap tailoring. Figure is available in color online only

**Fig. 4:**
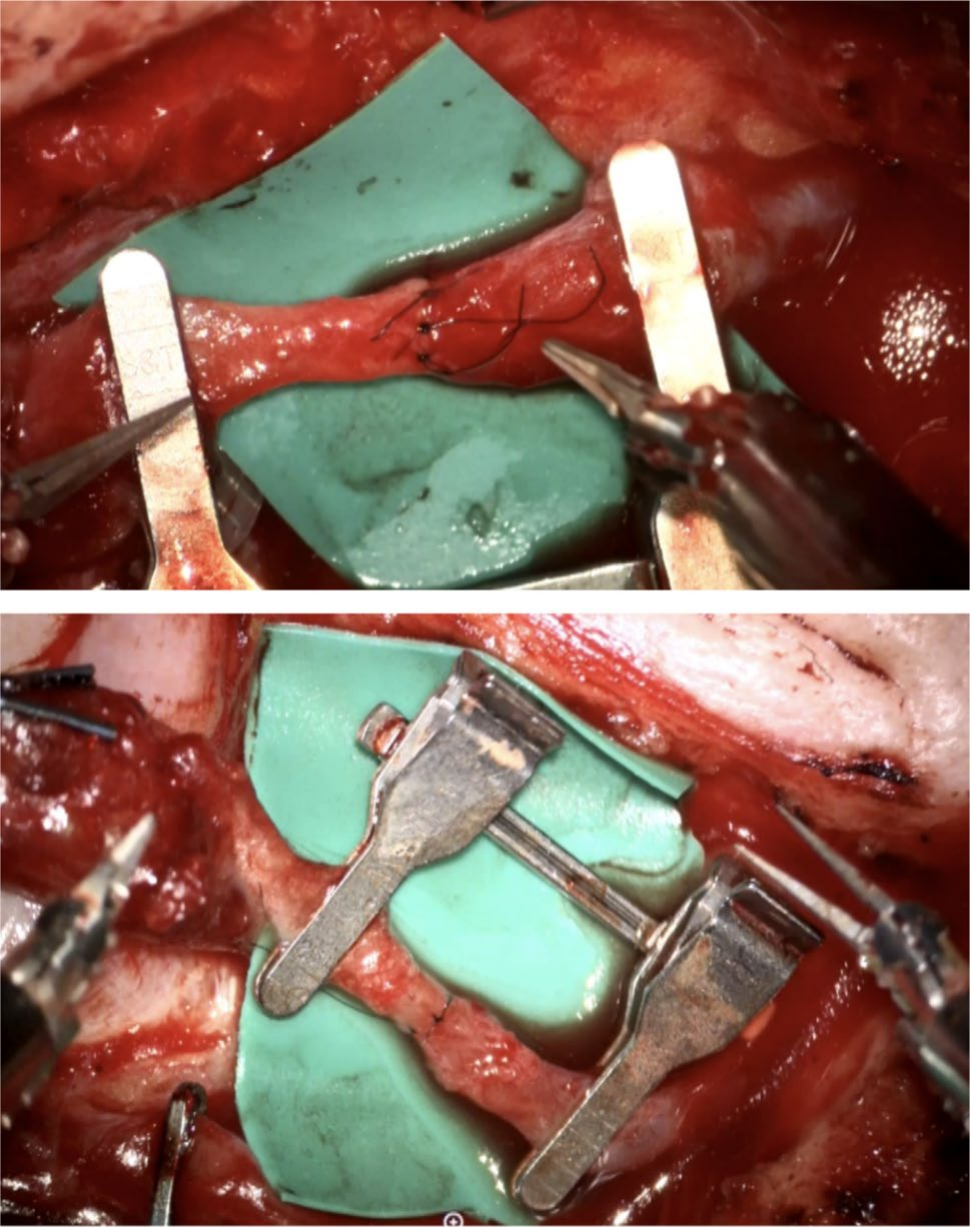
End-to-End anastomosis performed with the Symani^®^ Surgical System. The Symani^®^ Surgical System micro needle holder and microdilator allow precise stitch placement. Figure is available in color online only

In a total of 6 patients, 6 robotic-assisted End-to-End anastomoses were performed by the first two authors. Overall, 3 free ALT flaps and 3 free LD flap were transplanted to the scalp (Fig. 2, Fig. 3). In every arterial anastomosis, all knots were performed with the Symani^®^ Surgical System. Neither anastomosis required additional hand-sewn knots, all anastomoses were patent. Motion scaling was 7-10x. Patency of robotic-assisted anastomosis was 100% (Fig. 4). Sometimes due to regarding issues with the instruments, e.g. their becoming sticky from the blood which made them adhere or technical problems of the Symani^®^ Surgical System. This sometimes led to difficulty in suturing or troublesome cutting. The instruments needed to be cleaned through frequent rinsing or active help from the surgical assistant became necessary in some cases. Arterial anastomoses were analyzed by pulsation, manual flow control, Doppler examination, and flap perfusion.

The mean age was 71.8 years (range 61–81 years). 4 patients were male, and 2 patients were female. In 4 patients the STA was used as the recipient vessel. In 2 patients the FA was used as the recipient vasculature. A vein graft was never used. Mean time of the arterial anastomosis using the Symani^®^ Surgical System was 32.5 (19–44) minutes. Clinical and surgical data are reported in Table 1 and 2.

**Table 2.** Surgical data

Patient ID	Defect Location	infection signs recipient area	flap	Recipient Artery	Recipient Vein	Donor Site Skin Grafted	time operation (min)	time anastomosis (min)	Number of stitches with Symani/total	Suture size	additional procedures
1	Skalp (frontal)	Yes	ALT	Superficial Temporal	2 x Comitantes	No	339	33	9/9	9-0	PEEK plastic
2	Skalp (parietal)	No	LD	Superficial Temporal	Comitant	No	389	32	7/7	9-0	Frustrating flap lifting (ALT)
3	Skalp (frontal)	No	ALT	Superficial Temporal	2 x Comitantes	No	417	32	8/8	8-0	PEEK plastic
4	Skalp (frontal)	Yes	ALT	Facial	Facial + Comitant	No	438	35	8/8	9-0	PEEK plastic
5	Skalp (fronto-temporal)	No	LD	Facial	Facial	No	415	44	8/8	9-0	Apex scapulae graft
6	Skalp (fronto-parietal)	No	LD	Superficial Temporal	Comitant	No	328	19	7/7	9-0	PEEK plastic

^ALT=free anterolateral thigh flap; LD=free latissimus dorsi flap; PEEK=Polyether ether ketone^^; PAOD=peripheral arterial occlusive disease.^

Patient 1 underwent previously radiotherapy. Patients 3 and 5 underwent postoperative radiotherapy. Patients 1 to 4 underwent partial calvarias resection and reconstruction using PEEK plastics and titanium mesh respectively. There was no flap loss and no complication at the donor sites. There was no partial or total necrosis of the flaps. One complication was a PEEK infection and explanation again which occurred one month after flap reconstruction, followed by skull reconstruction with a Palacos^®^ plastic three months after flap reconstruction, but there was no flap related complication.

## Discussion

Overall, surgeons were able to definitively verify that the Symani^®^ Surgical System demonstrates a high degree of usability and precision in the application of microsurgical techniques. Our data show a safe and feasible system in free flap reconstruction of the scalp. The Symani^®^ Surgical System provides a high controllability and precision in placing the stitches. 3D-teleoperating with the device provides ergonometric conditions during the time of anastomosis, even in uncomfortable settings. This setting with the ability of teleoperation allows more space on the patient for an assistant or another operating team. In scalp reconstruction there is no limitation regarding positioning, the exposure of the STA or FA is very beneficial [1].

Importantly, despite these benefits, robotic microvascular surgery requires a highly skilled surgical team with extensive experience in microsurgery [7]. While robotic systems offer a series of advantages, they do not replace the basic microsurgical skills required for complex procedures. Successful integration of robotic technology depends on thorough training and proficiency in both traditional microsurgery and robotic techniques. In addition, surgeons must be prepared to deal with potential intraoperative challenges, such as equipment malfunctions or technical issues, to ensure that a safe transition to manual microsurgical techniques remains possible when needed. Therefore, the introduction of robotic microvascular surgery should be limited to experienced microsurgical teams in specialized centers. Another key aspect of robotic microvascular anastomosis is the duration of the procedure compared to the conventional hand-sewn technique. Currently, anastomosis times with the Symani® Surgical System tend to be longer, especially in the initial phase of implementation, due to system set-up requirements and the learning curve associated with robotic handling [16]. Yet, the aforementioned robotic advantages can contribute to more consistent suturing results and reduced fatigue during prolonged procedures. Our team recently found that robotic anastomosis times continue to decrease as surgeons become more experienced and optimized workflows are introduced [2]. Refinements such as improved docking efficiency, streamlined instrument changes, and greater familiarity with the robotic controls contribute to a more fluid surgical process. With continued training and adaptation, robotic anastomosis times could approach, and in some cases match, those of traditional techniques [2]. Future studies should further seek to quantify these improvements and evaluate whether robotic microsurgery can ultimately increase efficiency while maintaining or improving surgical outcomes.

A disadvantage of the Symani^®^ Surgical System is the absence of a touch sensation. We experienced a tendency towards longer anastomosis times. Other authors have also reported a significantly longer operation time when using the Symani^®^ Surgical System in head and neck reconstruction [13]. The longer duration in performing anastomoses did decline in time. Due to the magnetic field that translates the surgeon’s movements into the operative field, one can overcome the interference with the operating table by positioning the patient at the side of the operating table and using operating tables with as little metal as possible.

In 3 cases the STA was used as the recipient vessel. In 2 patients - due to STA-unavailability after previous neurosurgery in that field - the FA was used. In other studies, the STA/V was also the first-choice recipient vessel.

The ALT and LD flap are reliable options for reconstructions of large defects of the scalp by using the Symani^®^ Surgical System for microvascular anastomoses. Nowadays these flaps are the most common reconstructive flaps for scalp defect reconstructions and older age does not contraindicate the surgical procedure [7]. Both flaps can be easily tailored to the defect achieving satisfactory aesthetic outcomes. The ALT flap provides a smaller amount of tissue compared to the LD flap. Therefore, the ALT flap does not require any muscle tissue, but it can also be harvested with a part of the vastus lateralis muscle to fill up dead space or vascularized fascia lata to reconstruct the dura mater [7]. Pedicle length is variable. The ALT flap can provide a sufficient skin coverage, its thickness may vary and require a following debulking to optimize the shape. On the other hand, the anterolateral thigh flap can now also be raised in very thin layers [3]. The LD flap provides a large muscle and a long pedicle. A rib portion can repair skull defects. The LD flap requires split thickness skin grafting. Due to muscle atrophy, a contraction can result and can cause dehiscence’s. This can be avoided by harvesting larger flaps.

Free flap reconstruction of the scalp is indicated in large defects with or without skull prothesis or in which other reconstructions such as local flaps have failed. Also, in patients with insufficient tissue quality, preoperative or planned radiotherapy, full-thickness soft tissue defects and missing periosteum, free flap reconstruction is a sufficient option for wound closure. Free tissue reconstruction can provide an immediate closure of large defects and flap success rates are more than 95% [12]. When planning a reconstruction with a free flap one should always consider the patients conditions and goals in finding the ideal approach of reconstruction. There are a variety of free flap options, we report about our first cases in free flap reconstruction of the head with either a free ALT flap or a free LD flap using the Symani^®^ Surgical System for microvascular anastomosis.

Our study also revealed that robotic anastomotic may hold significant promise for neurosurgical bypass procedures, such as the superficial temporal artery to middle cerebral artery (STA-MCA) bypass. In this procedure, an extracranial donor artery (the STA) and an intracranial recipient artery (the MCA) are connected via an end-to-side microanastomosis using 10-0 microsutures [5]. This way the blood flow is rerouted from the extracranial to the intracranial circulation. Notably, the STA-MCA bypass is the most common neurosurgical bypass procedure, primarily used to augment blood flow to hypoperfused brain regions in patients with steno-occlusive disease of major cerebral arteries, to prevent ischemic stroke [14]. In addition, the STA-MCA bypass can serve as a flow replacement technique in the treatment of complex intracranial aneurysms, whereby the occlusion of an intracranial vessel is necessary [5]. In an STA-MCA bypass, the recipient artery is typically a superficial cortical branch of the MCA (M4-segment); hence, the microanastomosis is performed on the brain surface. This setting is particularly challenging due to the delicate and highly vulnerable nature of cortical vessels, the confined surgical field, and the need for absolute precision to avoid vascular injury, thrombosis, or brain damage.

The accuracy and stability of the Symani® Surgical System may enable neurosurgeons to perform such delicate microanastomoses with enhanced control, potentially reducing technical errors and minimizing vessel trauma. We believe that robotic assistance could allow neurosurgeons to perform the microanastomosis with high precision, control, and accuracy, as provided by the robotic arm. Still, while the preliminary results of robotic-assisted microanastomosis in plastic surgery are promising, it is important to recognize that further preclinical and clinical studies are required to evaluate the feasibility, efficacy, safety, and potential benefits of this approach in neurosurgical applications. We, therefore, are currently exploring the potential applications of robotic microsurgery in neurosurgery and anticipate that this innovative technology could represent a new frontier to be further investigated.

In this context, a key consideration in the future adoption of robotic microvascular surgery is its financial impact. Robotic systems, such as the Symani® Surgical System, require a significant initial investment, ongoing maintenance costs, and specialized (costly) surgeon training. These factors can pose an economic challenge, especially in smaller, less-resourced healthcare centers. However, potential benefits such as increased precision, improved ergonomics for the surgeon, shorter operating times, and potentially shorter hospital stays could contribute to cost-effectiveness over time. In addition, if robotic assistance leads to improved outcomes and fewer complications, this may further offset costs in the long run. Further studies with a thorough cost-benefit analysis are needed to determine the economic viability of robotic microvascular surgery in routine clinical practice.

We acknowledge that our study has inherent limitations, including the small sample size, its retrospective and single-center design, and the absence of a control group using conventional microanastomosis techniques. Our findings should, therefore, be interpreted with caution. However, it is important to note that our primary aim was to evaluate the feasibility and safety of the Symani® Surgical System for microvascular anastomosis in scalp reconstruction, rather than to directly compare it with traditional methods. Future prospective, multicenter studies with larger cohorts and control groups are needed to validate our results and further assess the clinical value of robotic-assisted microanastomosis in scalp reconstruction.

Our findings suggest that using the Symani^®^ Surgical System for arterial microanastomosis in head reconstruction is a safe and effective approach, leading to satisfactory outcomes. The system allows the successful execution of end-to-end anastomosis. The Symani^®^ Surgical System enables access to all anatomical regions of the head without limitations.

While the robotic assistance shows great potential, particularly with features like tremor filtering and motion scaling, further improvements in the grip of the microinstruments are necessary. The ability to perform precise movements, including the enhanced ergonomics, suggests an auspicious future for the integration of robotic systems in reconstructive microsurgery.

## Conclusion

Microvascular free flap reconstruction is the mainstay of large scalp defect reconstruction. Our group has previously demonstrated the feasibility and safety of using this robotic device for microsurgery in humans. This first series of robotic-assisted free flap transplantation to the scalp show the feasibility of the system. It is well known that microsurgery requires outstanding skills and extensive training. But there are subjective factors like tremor, precision, and accuracy that can be influenced by the physiological status of the surgeon. The Symani^®^ Surgical System can overcome these limits, and their performance is consistent without external influence. Therefore, it is a suitable option for microanastomosis in free flap reconstruction of the scalp.

## Data Availability

No datasets were generated or analysed during the current study.

## References

[1] Awwad L, Obed D, Vogt PM, Kaltenborn A, Koenneker S (2022) Superficial Temporal Recipient Vessels for Craniofacial Microvascular Free-Flaps. J Craniofac Surg 33:e652–e657. 10.1097/SCS.0000000000008768 35864586 10.1097/SCS.0000000000008768

[2] Barbon C, Grunherz L, Uyulmaz S, Giovanoli P, Lindenblatt N (2022) Exploring the learning curve of a new robotic microsurgical system for microsurgery. JPRAS Open 34:126–133. 10.1016/j.jpra.2022.09.002 36304073 10.1016/j.jpra.2022.09.002 PMC9593278

[3] Brown E, Suh HP, Han HH, Pak CJ, Hong JP (2020) Best New Flaps and Tips for Success in Microsurgery. Plast Reconstr Surg 146:796e–807e. 10.1097/PRS.0000000000007331 33234979 10.1097/PRS.0000000000007331

[4] Dastagir N, Obed D, Tamulevicius M, Dastagir K, Vogt PM (2024) The Use of the Symani Surgical System(R) in Emergency Hand Trauma Care. Surg Innov 31:460–465. 10.1177/15533506241262568 38884216 10.1177/15533506241262568 PMC11408963

[5] Esposito G, Amin-Hanjani S, Regli L (2016) Role of and Indications for Bypass Surgery After Carotid Occlusion Surgery Study (COSS)? Stroke 47:282–290. 10.1161/strokeaha.115.008220 26658449 10.1161/STROKEAHA.115.008220

[6] Gousopoulos E, Grünherz L, Giovanoli P, Lindenblatt N (2023) Robotic-assisted microsurgery for lymphedema treatment. Plastic and Aesthetic Research 10:7. 10.20517/2347-9264.2022.101

[7] Grünherz L, Gousopoulos E, Barbon C, Uyulmaz S, Giovanoli P, Lindenblatt N (2023) Robotics in plastic surgery. Chirurgie (Heidelb) 94:325–329. 10.1007/s00104-022-01790-w 36625922 10.1007/s00104-022-01790-w PMC10042931

[8] Grünherz L, Weinzierl A, Puippe GD, von Reibnitz D, Barbon C, Schneider MA, Giovanoli P, Gutschow CA, Lindenblatt N (2023) First-in-human Use of a Microsurgical Robotic System for Central Lymphatic Reconstruction. Plast Reconstr Surg Glob Open 11:e5484. 10.1097/gox.0000000000005484 38115836 10.1097/GOX.0000000000005484 PMC10730044

[9] Innocenti A, Menichini G, Innocenti M (2022) Six-years experience in major scalp defect reconstruction with free flap: analysis of the results. Acta Biomed 92:e2021301. 10.23750/abm.v92i6.10089 35075095 10.23750/abm.v92i6.10089 PMC8823577

[10] Innocenti M, Malzone G, Menichini G (2023) First-in-Human Free Flap Tissue Reconstruction Using a Dedicated Microsurgical Robotic Platform. Plast Reconstr Surg 151:1078–1082. 10.1097/prs.0000000000010108 36563175 10.1097/PRS.0000000000010108

[11] Könneker S, Watson JA, Weinzierl A, von Reibnitz D, Besmens I, Kim BS, Giovanoli P, Lindenblatt N (2025) Advances in Reconstructive Robotic Microsurgery in the Extremity. J Craniofac Surg 36:354–357. 10.1097/scs.0000000000010622 39283079 10.1097/SCS.0000000000010622

[12] Lamaris GA, Knackstedt R, Couto RA, Abedi N, Durand P, Gastman B (2017) The Anterolateral Thigh Flap as the Flap of Choice for Scalp Reconstruction. J Craniofac Surg 28:472–476. 10.1097/SCS.0000000000003404 28114212 10.1097/SCS.0000000000003404

[13] Lindenblatt N, Grünherz L, Wang A, Gousopoulos E, Barbon C, Uyulmaz S, Giovanoli P (2022) Early Experience Using a New Robotic Microsurgical System for Lymphatic Surgery. Plast Reconstr Surg Glob Open 10:e4013. 10.1097/gox.0000000000004013 35028251 10.1097/GOX.0000000000004013 PMC8747501

[14] Sebök M, Höbner LM, Grob A, Fierstra J, Schubert T, Wegener S, Luft AR, Kulcsár Z, Regli L, Esposito G (2025) Flow capacity of a superficial temporal artery as a donor in a consecutive series of 100 patients with superficial temporal artery-middle cerebral artery bypass. J Neurosurg 142:62–69. 10.3171/2024.4.Jns24247 39126722 10.3171/2024.4.JNS24247

[15] Sokoya M, Misch E, Vincent A, Wang W, Kadakia S, Ducic Y, Smith J (2019) Free Tissue Reconstruction of the Scalp. Semin Plast Surg 33:67–71. 10.1055/s-0039-1678470 30863215 10.1055/s-0039-1678470 PMC6408242

[16] Tolksdorf K, Hohberger FS, Ernst C, Tietz S, Schultze-Mosgau S, Tautenhahn F (2024) First experience using a novel microsurgical robotic device for free flap surgery in cranio- and maxillofacial surgery. J Craniomaxillofac Surg 52:704–706. 10.1016/j.jcms.2024.03.017 38627187 10.1016/j.jcms.2024.03.017

[17] van Mulken TJM, Wolfs J, Qiu SS, Scharmga AMJ, Schols RM, Spiekerman van Weezelenburg MA, Cau R, van der Hulst R, MicroSurgical Robot Research G (2022) One-Year Outcomes of the First Human Trial on Robot-Assisted Lymphaticovenous Anastomosis for Breast Cancer-Related Lymphedema. Plast Reconstr Surg 149:151–161. 10.1097/PRS.0000000000008670 34936615 10.1097/PRS.0000000000008670

[18] von Reibnitz D, Weinzierl A, Barbon C, Gutschow CA, Giovanoli P, Grünherz L, Lindenblatt N (2024) 100 anastomoses: a two-year single-center experience with robotic-assisted micro- and supermicrosurgery for lymphatic reconstruction. J Robot Surg 18:164. 10.1007/s11701-024-01937-3 38581589 10.1007/s11701-024-01937-3 PMC10998780

[19] Weinzierl A, Barbon C, Gousopoulos E, von Reibnitz D, Giovanoli P, Grunherz L, Lindenblatt N (2023) Benefits of robotic-assisted lymphatic microsurgery in deep anatomical planes. JPRAS Open 37:145–154. 10.1016/j.jpra.2023.07.001 37546233 10.1016/j.jpra.2023.07.001 PMC10403710

